# Bias of Medical Testimony Regulated by a Commission of Experts

**Published:** 1894-11

**Authors:** 


### Editorial.

### BIAS OF MEDICAL TESTIMONY REGULATED BY A COMMISSION OF EXPERTS.

"PROFESSIONAL Bias in Theory and Practice," is the title of an editorial, which appeared in the *Atlanta Medical and Surgical Journal* for Sept., 1884, and that is the key to these remarks.

When physicians and surgeons are called upon to give evidence in court by lawyers having an interest in a case, they are put in possession of the facts usually by a full and thorough examination of the injured party. Yet notwithstanding that the same opportunity is afforded these examiners on both sides of the suit, it is not uncommon to find quite an antagonism in the views of the medical witnesses secured by the opposing parties. Even matters of observation in the condition presented by the subject of such investigations make quite a different impression according to the standpoint of the observer and if a joint examination be made there is often a direct issue raised as to the facts of the case. It is very common for instance to find in an action for damages against a railroad company or other corporation that the surgeon of such party sees nothing in the injured subject which entitles him or her to damages; on the contrary, the surgeons employed by the prosecutor are prone to discern deformities and defects resulting from the injury which plainly indicate a claim to indemnity.

This attitude of medical witnesses can only be accounted for by some previous influence exerted upon their minds by their respective championship of the cause of the different parties to the suit. It must strike outsiders frequently that there is at the bottom of this difference in the views of doctors enlisted on opposite sides, some consideration of pecuniary interest. In so far as this may be well-founded, it must prove detrimental to the medical profession; and it is greatly to be desired that something shall be done to obviate this impression so prejudicial to the character of those who are brought forward as medical experts.

A plan has been proposed for the formation of a commission of medical men to act in such cases, and receive the statements of medical attendants or examiners from both sides, upon which a report shall be based for the guidance of the court and jury in damage suits. If the testimony of medical men is laid before such a tribunal there cannot be such a bearing towards either party as is frequently manifested in being catechised by the opposing attorneys. It is a legitimate inference that subjective and objective symptoms presented by the respective medical witnesses, would be thoroughly w ighed by the members of such a medical commission, and that a correct conclusion as to the case would be presented to the court. It is not, however, alone in damage suits that such a commission is indicated. The bearing of medical testimony as to the nature of wounds inflicted with murderous intent is often a grave consideration, and the bias due to the preconceived opinions of the doctor may influence materially the results of a legal investigation. The adjudication by a medical commission would tend to correct erroneous conclusions in all such cases.

Again, we have not infrequently in our courts of justice to determine upon the mental condition of one charged with criminal assault, and there is often a plea of insanity, when the question is raised as to its reality, which demands careful investigation. It so happens that in such a case medical men are arrayed in support of the State and the prisoner with diametrically opposite views of the responsibility of the prisoner, due to the influence brought to bear upon them by their relations to the respective parties. There is doubtless a sincere desire to arrive at a correct solution of the doubtful question in regard to the mental status of the subject, yet without charging any want of good faith upon either side, the discrepancy exists in their testimony, often having the same data for consideration.

There are very few general practitioners in the medical profession who are competent to arrive at the truth in regard to such unsoundness of mind as should render the subject free from responsibility, and much less to determine upon a case of feigned insanity, so that this variance in their views is a natural consequence of ignorance. When contradictory opinions are presented to the court by medical witnesses in regard to a case of insanity, it is most likely that a jury will lay no stres upon either view, and the verdict is worth nothing more than the decision by dice, or throwing up heads and tails to settle the question.

It is in such a contingency that a commission of experts is pre-eminently demanded, and notwithstanding the difficulty in securing competent members of the profession for this highly responsible service, the important consequences attending a correct decision in this class of cases warrants all the expense and trouble of providing such a commission.

It is not expected that each court should have a permanent commission of medical experts, but that the appointment of three members of the profession supposed to have the requisite qualifications for considering the special case, shall be made by the judge on the occasion of a trial before his court. Neither of those appointed ought to have any connection with the parties interested in the suit, and should receive compensation from the State or county, so as to occupy an independent position. An appointment of such a commission, with power to receive medical testimony under oath, and to make special examination of the subject of injury under investigation, must divest the proceedings of a court of many embarrassments growing out of the catechising of medical witnesses by the attorneys on opposite sides of a case. The ludicrous scenes enacted in court-houses by the questions propounded by lawyers, and the answers given by doctors, demonstrating the ignorance of both parties, calls for some such measure as a commission of medical experts for the trial of this class of cases, and it should be adopted by our courts at an early day.

J. M. F. G.

